# *Francisella novicida* and *F*. *philomiragia* biofilm features conditionning fitness in spring water and in presence of antibiotics

**DOI:** 10.1371/journal.pone.0228591

**Published:** 2020-02-05

**Authors:** Claire Siebert, Corinne Villers, Georgios Pavlou, Bastien Touquet, Nandadeva Yakandawala, Isabelle Tardieux, Patricia Renesto

**Affiliations:** 1 TIMC-IMAG UMR 5525—UGA CNRS, Grenoble Cedex 9, France; 2 Université de Caen Normandie, EA4655 U2RM, Caen, France; 3 Institute for Advanced Biosciences (IAB), Team Membrane Dynamics of Parasite-Host Cell Interactions, CNRS UMR 5309, INSERM U1209, Université Grenoble Alpes, Grenoble, France; 4 Kane Biotech, Inc., Winnipeg, Canada; Centre National de la Recherche Scientifique, FRANCE

## Abstract

Biofilms are currently considered as a predominant lifestyle of many bacteria in nature. While they promote survival of microbes, biofilms also potentially increase the threats to animal and public health in case of pathogenic species. They not only facilitate bacteria transmission and persistence, but also promote spreading of antibiotic resistance leading to chronic infections. In the case of *Francisella tularensis*, the causative agent of tularemia, biofilms have remained largely enigmatic. Here, applying live and static confocal microscopy, we report growth and ultrastructural organization of the biofilms formed *in vitro* by these microorganisms over the early transition from coccobacillary into coccoid shape during biofilm assembly. Using selective dispersing agents, we provided evidence for extracellular DNA (eDNA) being a major and conserved structural component of mature biofilms formed by both *F*. subsp. *novicida* and a human clinical isolate of *F*. *philomiragia*. We also observed a higher physical robustness of *F*. *novicida* biofilm as compared to *F*. *philomiragia* one, a feature likely promoted by specific polysaccharides. Further, *F*. *novicida* biofilms resisted significantly better to ciprofloxacin than their planktonic counterparts. Importantly, when grown in biofilms, both *Francisella* species survived longer in cold water as compared to free-living bacteria, a trait possibly associated with a gain in fitness in the natural aquatic environment. Overall, this study provides information on survival of *Francisella* when embedded with biofilms that should improve both the future management of biofilm-related infections and the design of effective strategies to tackle down the problematic issue of bacteria persistence in aquatic ecosystems.

## Introduction

*Francisella tularensis* is a non-motile Gram-negative coccobacillus and the causative agent of zoonotic tularemia disease. Due to a remarkable infectivity (< 10 bacteria) which is associated with high mortality and morbidity and a good genetic tractability, the US Centers for Disease Control and Prevention (CDC) has classified *F*. *tularensis* as category A bioterrorism agent [1]. The ability of *Francisella* strains to form biofilms *in vitro* was first reported in 2009 with *F*. *novicida* [2] and later observed with other *Francisella* species including highly virulent [3] and environmental strains [4]. Given the importance of biofilms in infectious diseases [5–10], the regulation of *Francisella* biofilm assembly was investigated and led to identifying the Sec secretion system [3] as well as of the transcription factor *qse*B [11] as main players of the biofilm regulatory cascade [12]. Although not demonstrated experimentally, and considering the role of *Francisella* chitinases in biofilm formation, it has been hypothesized that biofilms would significantly contribute to *Francisella* persistence in both aquatic habitats and mosquito hosting vectors [3, 13, 14]. The correlation between *Francisella* biofilm dispersion and an increased bacterial susceptibility towards biocides has been reported [14, 15] and we have recently brought first genetic evidence that the biofilm growth mode provides *F*. *tularensis* with a reduced susceptibility towards fluoroquinolones [16].

Tularemia is associated with several clinical forms whose severity depends not only on the *Francisella* subspecies, but also on the route of infection [17]. The more severe disease in human is caused by inhalation of *F*. *tularensis* subsp. *tularensis* (Type A) leading to a pneumonic form of tularemia [18]. Although the epidemiology of this disease is still not fully understood, risk factors include exposure to arthropods, especially ticks which are the most important vectors of the terrestrial life cycle of Type A *Francisella* [1, 19]. The two unusual outbreaks of pneumonic tularemia reported from the island of Martha’s Vineyard [20, 21] together with the observed prolonged survival of Type A *F*. *tularensis* in brackish-water collected on this island [22] allowed hypothesizing that the aquatic environment could serve as a reservoir for *F*. *tularensis* [13, 23]. *F*. *tularensis* subsp. *holarctica* (Type B) being widespread in the Northern hemisphere is the other subspecies of major clinical importance although the disease is rarely fatal in humans [24]. Clinical and epidemiological data have highlighted a close relationship between outbreaks of *F*. *tularensis* subsp. *holarctica* with water sources while infected mosquitoes were described as major vectors of these bacteria, in particular in Scandinavia [1, 25–27]. *F*. *tularensis* subsp. *novicida* (herein *F*. *novicida*) is described as a rare opportunistic human pathogen despite being responsible for severe pathologies in either immuno- or medically weakened patients [28]. In healthy individuals, the consumption of contaminated water was identified as a major source of acute *F*. *novicida* infection following near-drowning events and ingestion of icy water [29–31]. Being the most genetically tractable *Francisella* subspecies, *F*. *novicida* has largely been used as an experimental relevant model to study *F*. *tularensis* pathogenesis [28, 32, 33]. *F*. *philomiragia* (previously *Yersinia philomiragia*) is another *Francisella* species which is commonly found in soil, water and aerosol samples [34]. While only a few cases of human infection with *F*. *philomiragia* have been described, they correlated with pneumonia in healthy individuals undergoing near-drowning accidents or in immunocompromised people. Overall, water appears as an important reservoir for some human pathogenic *Francisella* strains [23].

To gain insights on the development and composition of *Francisella* biofilms, a prerequisite for designing anti-biofilm efficient strategies to target the bacteria biofilms, we compared the main features of the biofilm dynamics and composition for both *F*. *novicida* and F. *philomiragia*. *F*. *novicida* strain U112 was used as relevant model for the highly pathogenic *Francisella* subsp. *tularensi*s [28] and the *F*. *philomiragia* strain CHUGA-FP47 isolated from a patient [35] was used as a clinically relevant isolate. We visualized in real time the biofilm formation and analyzed the extracellular polymeric substances (EPS) of mature biofilms using selective dispersing agents. Further, the behavior of both planktonic and biofilm forms of *Francisella* was assessed both under antibiotics and cold water exposure.

## Materials and methods

### Bacterial strains and growth conditions

*F*. *novicida* (U112) CIP56.12 (Centre de Ressources Biologiques de l'Institut Pasteur, Paris, France) and *F*. *philomiragia* (CHUGA-FP47) were grown either on solid or in liquid media, as indicated. The solid medium corresponds to pre-made Polyvitex-enriched chocolate agar (PVX-CHA) plates (Ref#43109 BioMérieux, France) incubated at 37°C in a 5% CO_2_ incubator with humidified atmosphere and used both to revive glycerol stocks and for CFU counting. Liquid cultures were carried out in Modified Mueller-Hinton broth (MMH) at 37°C under shaking. MMH corresponds to Mueller Hinton Broth (Ref#275730 Grosseron) supplemented with NaCl (Sigma; 5 g/L), BactoProteose peptone (Ref#211693 BD Biosciences; 5 g/L), BactoTryptone (Ref#211705 BD Biosciences 5 g/L) and L-cysteine (Ref#C7477 Sigma; 1 g/L).

### Biofilm quantification

The static biofilm was grown in 96-well plates and detected by crystal violet staining [36]. Briefly, liquid bacterial cultures (absorbance around 0.8 at OD_600 nm_) were diluted in 37°C pre-warmed MMH and 200 μL of bacterial suspensions were dispensed per well of flat-bottom polystyrene 96-well plates (Falcon®). To avoid the edge effect resulting from evaporation, the peripheral wells were filled with 200 μL PBS. Plates were incubated for 24 h to 72 h at 37°C in a humidified atmosphere containing 5*%* CO_2_. The OD_600 nm_ was measured using Tecan Plate reader to normalize bacterial growth and planktonic bacteria were gently aspirated. *F*. *novicida* biofilms were washed twice with sterile PBS while *F*. *philomiragia* biofilms were washed only once to ensure minimum to negligible amount of loss of bacteria as assessed by count of the colony forming unit (CFU) from washings. Microtiter plates were then incubated for 1 h at 70°C, stained with 200 μL of 0.1% (w/v) crystal violet/well (Merck) for 15 min and washed twice with 200 μL H_2_O. Biofilms were solubilized in 200 μL 30% acetic acid per well and quantified by measuring optical density at 595 nm.

### Fluorescence microscopy

For imaging, 1 mL of bacteria in MMH (1x10^7^/mL for *F*. *novicida* and 5x10^7^/mL *F*. *philomiragia*) were distributed in 24-well plates containing poly-L-lysine-coated glass coverslips. Samples were fixed at different time points with freshly made 4*%* paraformaldehyde in PBS for 20 minutes at room temperature. The supernatant containing planktonic cells was gently removed allowing the biofilm at the air-liquid interface to adhere to the coverslip. The wells were washed twice with PBS to remove residual planktonic cells and incubated in 50 mM NH_4_Cl in PBS for 5 min to quench auto-fluorescence caused by free aldehydes. Samples were stored in PBS supplemented with 0.02% (w/v) sodium azide at 4°C until staining. For staining of both bacterial membranes and biofilm glycoproteins, fixed samples were incubated in 5 μg/mL FM^®^1-43FX membrane probe (Life Technologies) and 200 μg/mL concanavalin (ConA)-FITC (Life Technologies) for 30 min at RT and washed 3 times with PBS. Nucleic acids were then stained with 0.5 μg/mL DAPI fluorescent dye for 5 min and the coverslips were mounted in Mowiol mounting medium. In some experiments, the presence of eDNA was visualized on unfixed biofilms and using the cell-impermeant DITO^™^-1 (2 μM; AAT Bioquest), a chemical analog to TOTO® together with Hoechst 33342 (1:5,000). The viability of bacteria within biofilms was also assessed from unfixed samples stained for 30 min at room temperature with the membrane impermeant dye propidium iodide (PI) (3 μM, Molecular Probes) that does not penetrate intact cells. Slides were imaged under Zeiss Apo Tome microscope with 63x/1.4 oil-immersion objective using Zeiss Zen software. Images were further processed using ImageJ [37] and Adobe Photoshop software. 3D reconstruction of images was achieved by processing raw data (i.e., xyz files) using ImageJ software to crop the sequence and region of interest. When needed, z stack from each channel was deconvoluted using the “Iterative Deconvolve 3D” plugin with a z-step of 0.3 μm. Zen software was used for 3D reconstruction and to produce movies from processed images.

### Time-lapse microscopy and image processing

Time-lapse video microscopy was performed with bacteria diluted in MMH and placed in Chamlide^™^ chambers (LCI Corp., Seoul, Korea) installed on an Eclipse Ti inverted confocal microscope (Nikon France Instruments, Champigny sur Marne, France) with a temperature (37°C) and CO_2_ (5%)-controlled stage. Images were collected every 15 minutes with a z-step of 0.3 μm using a CMOS camera (Photometrics, Tucson, AZ, USA) and a CSU X1 spinning disk (Yokogawa, Roper Scientific, Lisses, France). Analysis was performed using MetaMorph software (http://www.moleculardevices.com) from the raw image data files.

### Dispersion of *Francisella* biofilms

The effect of dispersants was evaluated by using 24 h and 48 h-old biofilms of *F*. *novicida* and *F*. *philomiragia* biofilms, respectively. *F*. *novicida* biofilms were grown in 96-well polystyrene plates (Falcon^®^) starting from an inoculum of 1x10^7^ CFU/mL (2x10^6^ bacteria/well), while for *F*. *philomiragia* 5x10^7^ CFU/m (10^7^ bacteria/well) were used. After one (*F*. *philomiragia*) or two (*F*. *novicida*) gentle PBS washes, the MMH was replaced with 200 μL fresh medium supplemented with increasing concentrations of dispersing agents including DNase I (Roche), EDTA (Sigma-Aldrich), chitinase from *Streptomyces griseus* (E.C. 3.2.1.14) (Sigma-Aldrich), cellulase (Sigma-Aldrich), proteinase K (Invitrogen) or Dispersin B (Kane Biotech Inc. Canada). Microtiter plates were incubated at 37°C for 24 h under 5% CO_2_ and biofilm was quantified as described elsewhere. Data were expressed as percentage of remaining biofilm calculated in comparison to untreated biofilm under the same experimental conditions.

### Evaluation of antimicrobial resistance of planktonic and biofilm bacteria

The antibiotic resistance of *F*. *novicida* and *F*. *philomiragia* was evaluated under the same conditions as the dispersion assays but using MMH supplemented with increasing concentrations of antibiotics. Following 24 h incubation at 37°C the bacterial viability was evaluated using both the resazurin assay detailed above and the CFU method, as previously described and detailed below [16]. The antibiotic susceptibility of planktonic bacteria was assessed in parallel using the same culture medium and starting from a bacterial suspension containing an equal amount of bacteria as those present in biofilms. The growth of planktonic bacteria was evaluated by OD_600nm_ measurement and their viability was tested by the metabolic rezasurin test (see below). The minimal inhibitory concentrations (MICs) of gentamicin (Panpharma) and ciprofloxacin (Sigma-Aldrich) used as reference values were obtained using the broth-dilution method and in line with the CLSI guidelines [38]. For *F*. *novicida*, they were of 1 mg/L and 0.064 mg/L, respectively, these values being of 0.5 mg/L and 0.032 mg/L for *F*. *philomiragia*,

### Survival of planktonic or biofilm bacteria in water

*F*. *novicida* and *F*. *philomiragia* biofilms were grown and washed as described for dispersal experiments. After washings, the wells were filled with 200 μL of water collected from a spring around Grenoble and sterilized by filtration using 0.45 μm then 0.22 μm filters. The microtiter plates were stored at 4°C, and bacteria survival was assessed by CFU assay at each time point. In parallel, the viability of planktonic bacteria was determined starting from exponential growth phase bacteria. The bacterial suspension was centrifuged and washed with PBS and the resulting pellet was resuspended in a volume of spring water such as the bacterial density was close to that found within biofilms (5x10^7^ bacteria/200 μL).

### CFU counting

Biofilms previously disrupted to release cells by vigorous pipetting with PBS and planktonic cell suspensions were serially diluted in PBS. For each sample 100 μL of at least four different dilutions were plated on PVX-CHA plates, incubated for 24–48 h at 37°C under 5% CO_2_, and CFU were counted.

### Measurement of bacterial viability by resazurin assay

This assays was done with resazurin (Sigma-Aldrich), a fluorescent indicator of mitochondrial function conveniently used for the evaluation of cell viability in of several bacterial species [39, 40] including *Neisseria gonorrhoeae* [41] and *Francisella* LVS strains [16]. In this metabolic assay, the incubation time was significantly lower (1–2 h *vs* 24h) than the conditions for which this compound was depicted as being an antimicrobial agent towards *Francisella* and *Neisseiria* species [42]. Viable bacteria reduce blue non-fluorescent resazurin dye (λ_max_ = 600 nm) to the pink fluorescent compound resorufin (max absorbance λ_max_ = 570 nm). Antibiotic treated or untreated biofilms were washed with PBS to remove planktonic cells, supplemented with 200 μL/well of a 0.02 mg/mL resazurin (Sigma-Aldrich) solution in MMH, and incubated for 2 h at 37°C under static conditions. Cell viability was estimated by using absorbance was measured at (OD_570nm_-OD_600nm_). Planktonic cells (200 μL) were supplemented with 20 μL of resazurin (0.2 mg/mL), incubated for 1 h at 37°C, and cell viability was evaluated and expressed as percent viability in comparison to untreated cells.

### Statistical analysis

Statistical analysis was performed using the GraphPad PRISM software and Student’s t- tests. Data correspond to means ± standard errors of the means (SEM) of at least three independent replicates from independent experiments, as depicted. *P* values less than 0.05 were considered statistically significant.

## Results

### Quantification of biofilm formation by *F*. *novicida* and *F*. *philomiragia*

**Fig 1:**
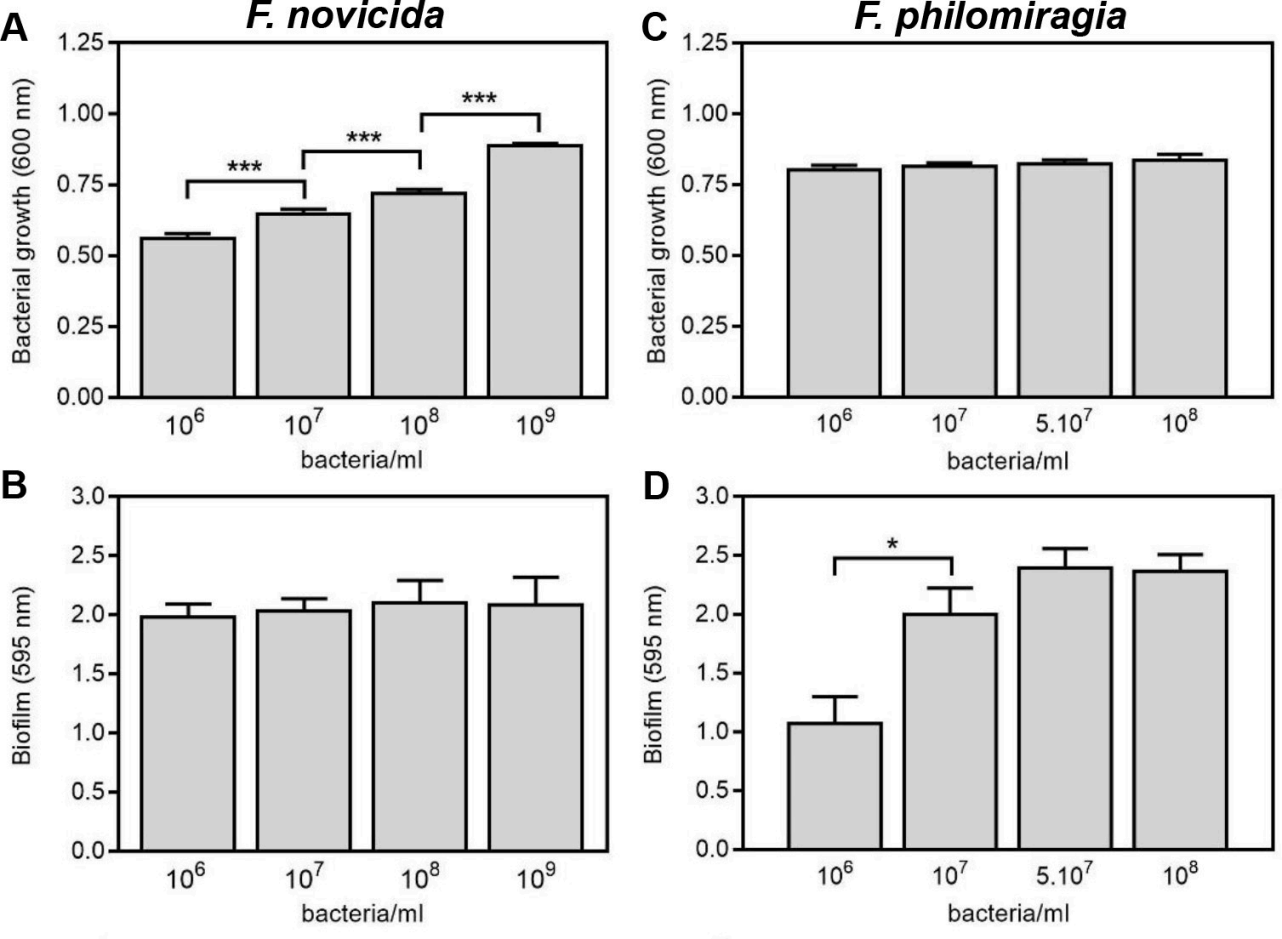
Quantitative measurement of biofilm formation by *F*. *novicida* and *F*. *philomiragia*. Biofilm formation was quantified in 96-well plates inoculated with increasing concentrations of bacteria and incubated under static conditions. Following 24 h (*F*. *novicida*) or 48 h (*F*. *philomiragia*) incubation at 37°C the bacterial growth **(A, C)** was quantified by OD_600nm_ and biofilm biomass **(B, D)** was estimated by crystal violet staining. The data correspond to the mean ± SEM of three or more independent experiments with *n* = 6 replicates for each. *P<0.05 and *** *P*<0.0005.

The ability of *F*. *novicida* U112 and *F*. *philomiragia* strain CHUGA-FP47 to produce biofilm was first compared starting from increasing concentrations of the bacterial inoculum incubated under static conditions in MMH broth at 37°C. The biofilm biomass was quantified by crystal violet staining, a basic dye that binds non-specifically to negatively charged surface including polysaccharides and eDNA present into the extracellular matrix. As shown Fig 1A, increasing *F*. *novicida* inoculum size translated in a higher bacterial density in the wells after a 24 h incubation time, while the biofilm biomass remained unchanged (Fig 1B). The largest amount of biofilm was observed for an inoculum of 10^6^−10^7^ bacteria/mL while its production estimated by the ratio biofilm biomass/bacteria number was seen to significantly drop beyond 10^8^ bacteria/mL (*P<0*.*05; n =* 12). The recovery of viable bacteria evaluated by CFU counting from a 24 h biofilm formed with 10^7^ bacteria/mL in the starting starting inoculum was of 4.95 x10^7^ ± 0.7x10^7^ bacteria/well (*n* = 13). While the *F*. *novicida* biofilm biomass measured after 48 h incubation was close to that observed at 24 h (not shown), the number of recovered bacteria was significantly increased 8.15 x10^7^ ± 2.4 x10^7^ bacteria/well (*n =* 4). In contrast to *F*. *novicida*, the *F*. *philomiragia* 24 h-old biofilm was found particularly fragile since it detached easily from the bottom of the microtiter plate and sheared despite gentle washing procedures (not shown). The quantification of *F*. *philomiragia* biofilm formation was thus analyzed over a 48 h incubation period. Under such conditions, the optimal inoculum for the *F*. *philomiragia* CHUGA-FP47 strain was of 5x10^7^ bacteria/mL (Fig 1C and 1D) leading to a recovery of 5.49 x10^7^ ± 1.38 x10^7^ viable bacteria from a 96 well-biofilm (*n* = 12).

### Visualization and microscopic analysis of *Francisella* biofilm growth

**Fig 2:**
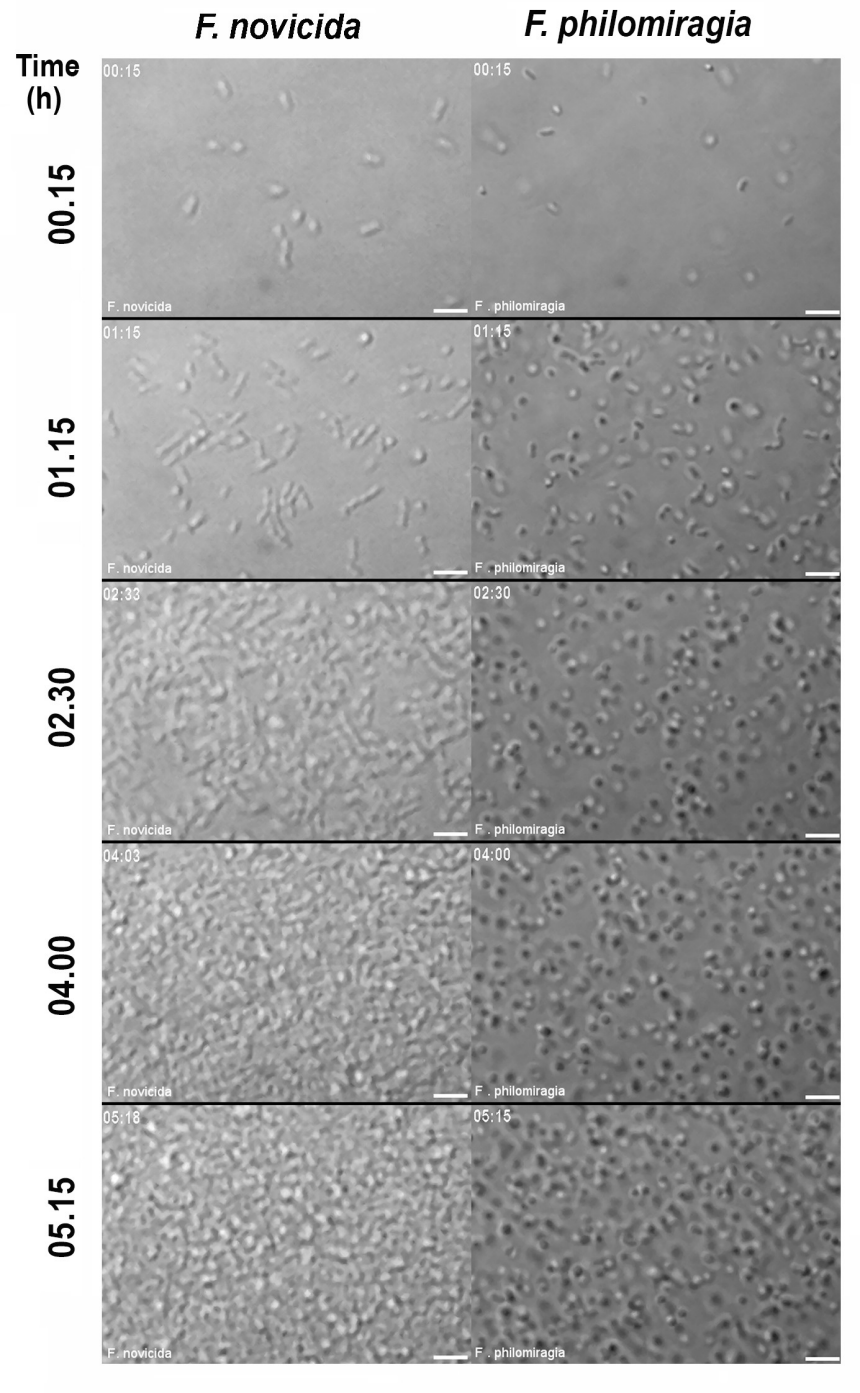
Kinetics of biofilm formation by *F*. *novicida* and *F*. *philomiragia*. Confocal images were taken at each of indicated time points from bacterial suspension grown at 37°C without agitation in Chamlide^™^ chambers installed on an Eclipse Ti inverted confocal microscope as detailed in Materials and Methods. The scale bar indicates 5μM.

**Fig 3:**
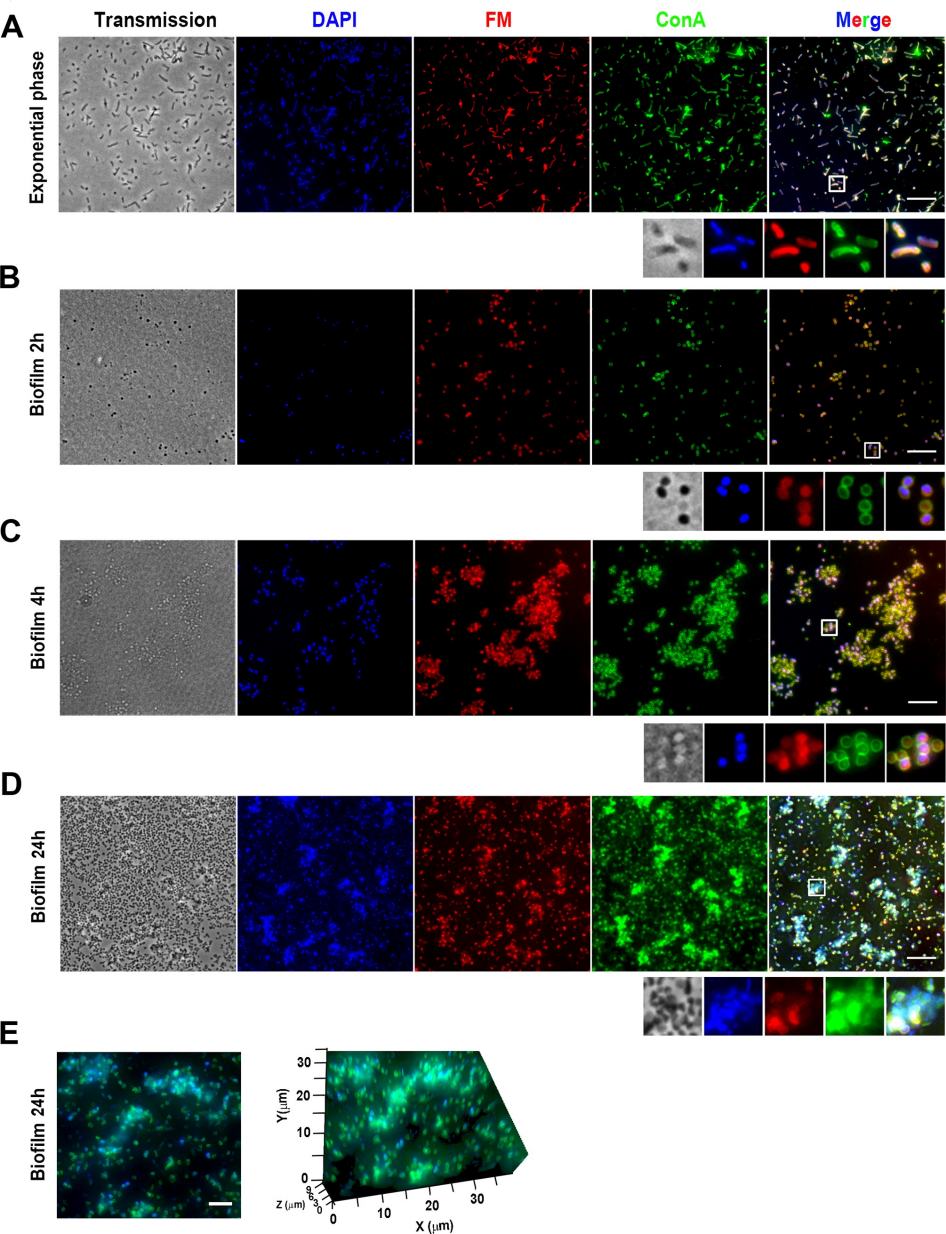
Fluorescence microscopy analysis of *F*. *novicida* biofilm formation. **(A)** Planktonic exponential phase bacteria and **(B-D)** biofilms formed upon incubation of bacteria for different time intervals were fixed and stained with DAPI (nucleic acids—blue), FM^®^1-43FX (FM; bacterial membranes—red), ConA-FITC (ConA; EPS sugar residues—green). The scale bar represents 10 μm. **(E)** Z maximal projection of the 24 h biofilm. The zoomed panels show the volume of the region of interest under two different orientations; note that the thickness of the biofilm reaches approximately 9 μm.

**Fig 4:**
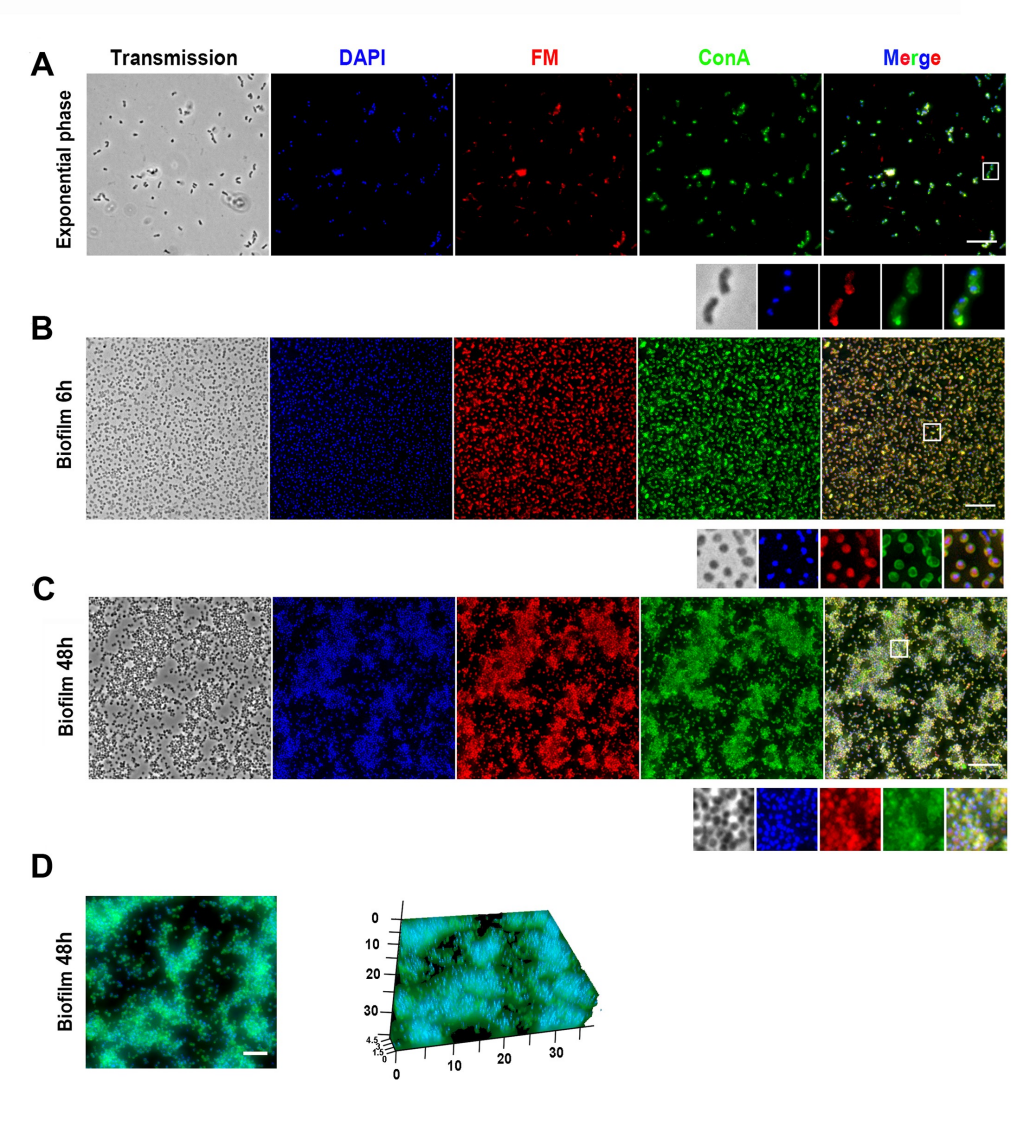
Fluorescence microscopy analysis of *F*. *philomiragia* biofilm formation. Same legend as in Fig 3 with a biofilm thickness at 48 h around 4.5 μm.

The formation of both *F*. *novicida* and *F*. *philomiragia* biofilms was then monitored during several hours by real time confocal laser scanning microscopy (CLSM) at 37°C (Fig 2 and S1 Movie). These observations confirmed the greater capacity of *F*. *novicida* to replicate and to spatially organize as biofilms. The biofilm evolution over time was also imaged using Structured Illumination Microscopy SIM (Figs 3 and 4). Over the 15 min post-inoculation period, no bacteria were observed on the coverslips used to retain the floating biofilm formed at the air-liquid interface after gentle aspiration of planktonic cells. To have a reference point prior to biofilm formation, we examined the bacterial suspension from the inoculum. For both strains, bacteria displayed an uniform coccobacillary shape visualized by the dual labeling of membrane and surface-exposed carbohydrates using the styryl fluorescent dye and the ConA lectin, respectively (Figs 3A and 4A). Over the few following hours, confocal images attest from cell-to-cell juxtaposition of bacteria secreting large amounts of glycoconjugates (Fig 3B–3E and Fig 4B–4D). The floating colonies progressively developed in a more robust biofilm with bacteria embedded in EPS. A striking morphological feature of both strains was the spherical shape they adopted in course of the biofilm formation, an effect observed as early as the 2 first hours for *F*. *novicida* (Fig 3B). During the 24 h of biofilm assembly, these bacteria continued to proliferate within the matrix and the thickness was estimated around 9 μm (Fig 3E). In agreement with the biomass quantification data, a delayed biofilm assembly was observed for *F*. *philomiragia*, with packed bacteria communities that clustered within a ~ 4.5 μm-thick EPS after 48 h (Fig 4D).

### Treatment of mature *Francisella* biofilms with potential dispersing agents

**Fig 5:**
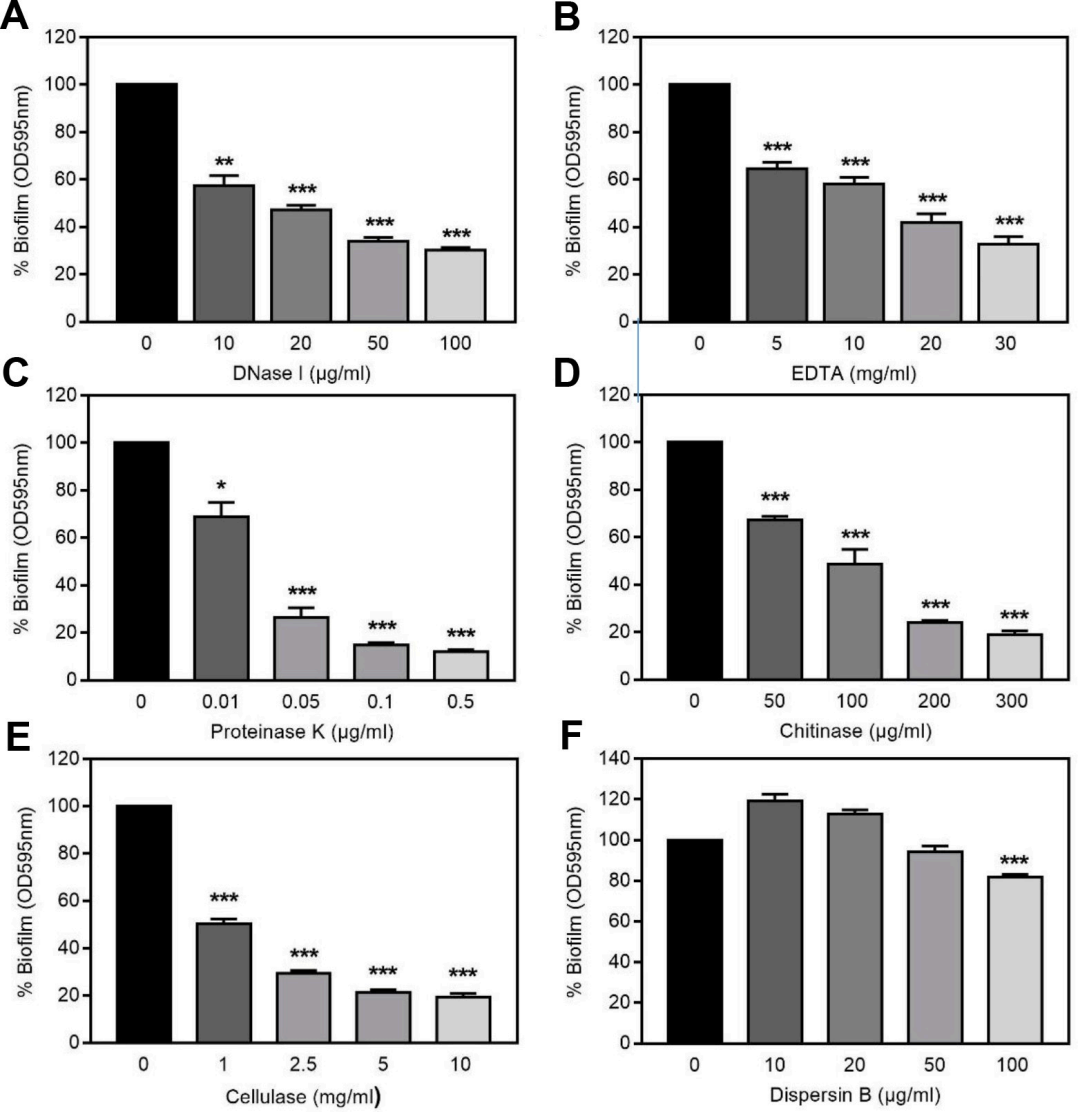
Disruption of *F*. *novicida* biofilms. *F*. *novicida* biofilms (24 h-old) were incubated with different concentrations of **(A)** DNase I, **(B)** EDTA, **(C**) proteinase K, **(D)** chitinase, **(E)** cellulase and **(F)** dispersin B. After 24 h incubation at 37°C, biofilm biomass was estimated by crystal violet staining. Values were expressed as percentage of data obtained in absence of dispersing agents and correspond to mean ± SEM from at least three different experiments with four replicates for each. * *P*<0.05, **P<0.005 and ***P<0.0005.

Given that main components of the bacterial biofilm EPS include eDNA, proteins and polysaccharides which adhere to each other and maintain microbial cells together [6], the capacity of enzymes or reagents targeting these specific compounds to induce biofilm dispersal was investigated. Taking in consideration biofilm biomass, thickness and the number of biofilm embedded bacteria, these assays were carried out using 24 h and 48 h-old biofilms of *F*. *novicida* and *F*. *philomiragia*, respectively. Results obtained showed that, following 24 h exposure, the DNase I induced a concentration-dependent dispersion of *F*. *novicida* biofilm with a maximum level of around 70% reached at 50 μg/mL (Fig 5A). The role of eDNA in *F*. *novicida* biofilm stabilization was further suggested using the chelating agent EDTA (Fig 5B) that similarly affected the structural integrity of the EPS matrix but by impacting the electrostatic interactions between divalent cations and negatively charged DNA [43]. Biofilm dispersion was almost complete (87.93% ± 0.8%; *n* = 12) following treatment with 0.5 μg/mL proteinase K, indicating that proteinaceous structures largely contribute to *F*. *novicida* biofilm structure and integrity (Fig 5C). This biofilm also contains polysaccharides and more specifically poly-β(1,4)-N-acetylglucosamine hydrolyzed by chitinase, leading to more than 80% dispersion when used at 300 μg/ml (Fig 5D). Another polysaccharide identified as a key component of *F*. *novicida* biofilm is the ß(1,4)-linked D glucose homopolymer cellulose (Fig 5E) which displays the same coupling as the acetylamine group with chitin. In contrast, dispersin B which targets poly-β-(1,6)-linked N-acetylglucosaminoglycans [44, 45] rather induced a moderate decrease of the biofilm biomass (Fig 5F). The capacity of DNase I, proteinase K and cellulase to dissociate *F*. *novicida* biofilms has been reported but under incubation time and concentration conditions different from our assays [14].

**Fig 6:**
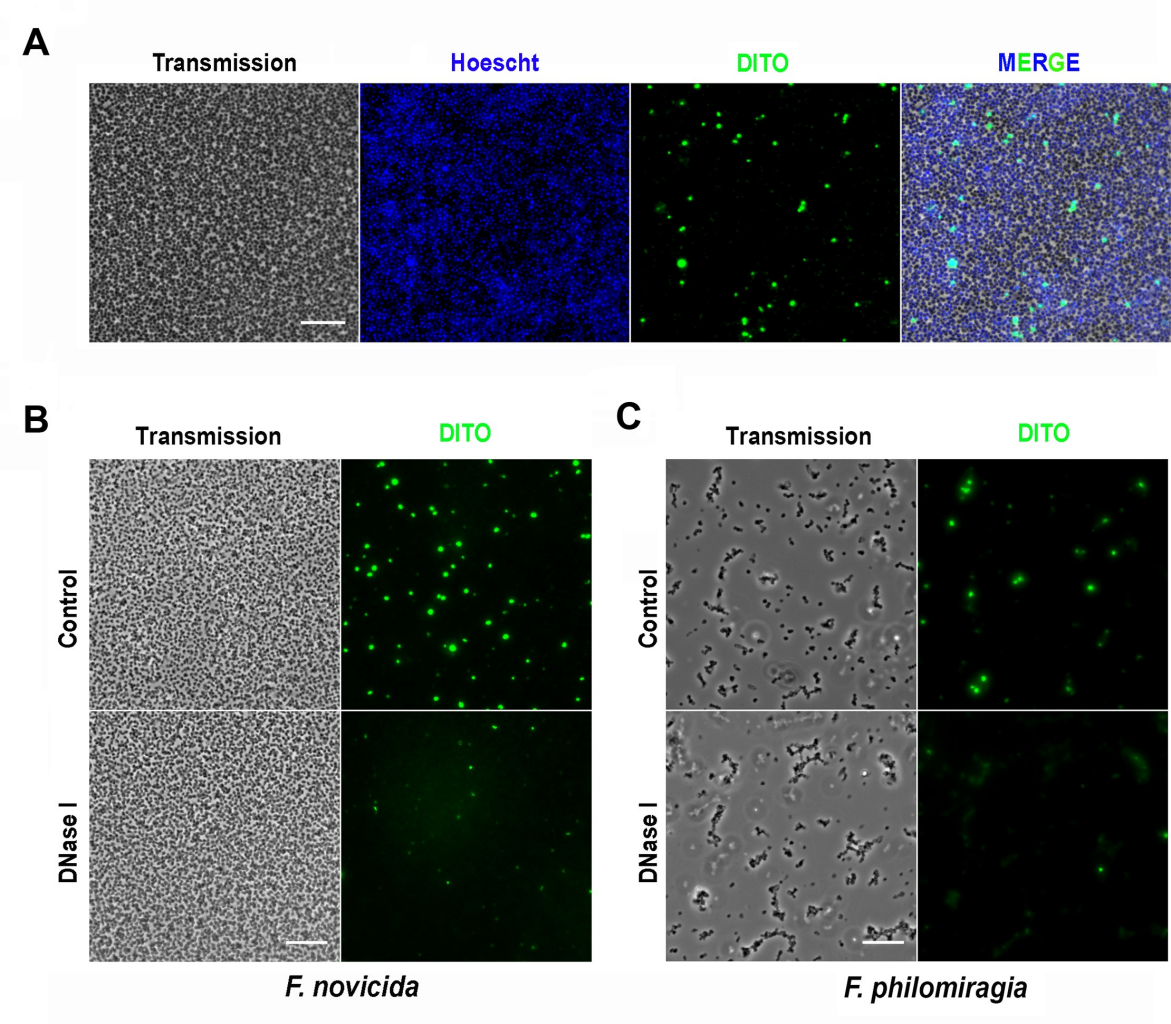
Evidence of eDNA in *Francisella* biofilms. **(A)** Brightfield and fluorescence images showing typical biofilm of *F*. *novicida* after 24 h growth. Whole bacterial population was stained with Hoescht (blue) and eDNA was visualized using DITO^TM^-1, a cell-impermeant, high-affinity nucleic acid stain (green). **(B)** Staining of 24 h-old *F*. *novicida* and **(C)** 48 h-old *F*. *philomiragia* biofilms following 1 h incubation with 100 μg/mL DNase 1 (lower panels) which efficiently reduced the amount of detected eDNA. The scale bars correspond to 10 μm.

Interestingly, the composition of the biofilm matrix appeared strikingly different for *F*. *philomiragia* (Table 1). Importantly, proteinase K did not impact the *F*. *philomiragia* biofilm biomass, even used at a concentration as high as 200 μg/mL, whereas dispersin B treatment led to a greater biofilm detachment than observed toward *F*. *novicida*. Taking into account these data together with the chitinase and cellulase inefficiency seen on *F*. *philomiragia* biofilm underscores the differences in EPS-secreted polysaccharides between both bacterial species. In contrast, and as observed with *F*. *novicida*, both DNase I and EDTA treatment significantly affected the biofilm formed by *F*. *philomiragia*, albeit at a lesser extent. Accordingly, eDNA seems to be a common and essential constituent of *Francisella* EPS, a point further confirmed from biofilms treated or not with DNase I prior staining and imaging using the selective DITO^™^-1 eDNA marker (Fig 6).

**Table 1:** Effect of various components on *F*. *novicida* and *F*. *philomiragia* biofilms.

Dispersant	Concentration	% of remaining biomass after treatment		*P* value
		*F*. *novicida*	*F*. *philomiragia*	
DNase I	100 μg/mL	30.2 ± 1.1	60.8 ± 4.4	0.0001
EDTA	30 mg/mL	32.8 ± 3.3	50.7 ± 3.3	0.0005
Proteinase K	200 μg/mL	12.2 ± 2.1	116.3 ± 6.9	0.0001
Chitinase	300 μg/mL	18.9 ± 1.7	104.2 ± 5.7	0.0001
Cellulase	50 mg/mL	19.9 ± 0.9	121.1 ± 10.7	0.0001
Dispersin B	100 μg/mL	81.7 ± 1.4	49,02 ± 3.6	0.0005

Same legend as in Fig 5 using 24 h and 48 h-old biofilms for *F*. *novicida* and *F*. *philomiragia*, respectively.

### Antimicrobial susceptibility of planktonic and biofilm-embedded *Francisella*

**Fig 7:**
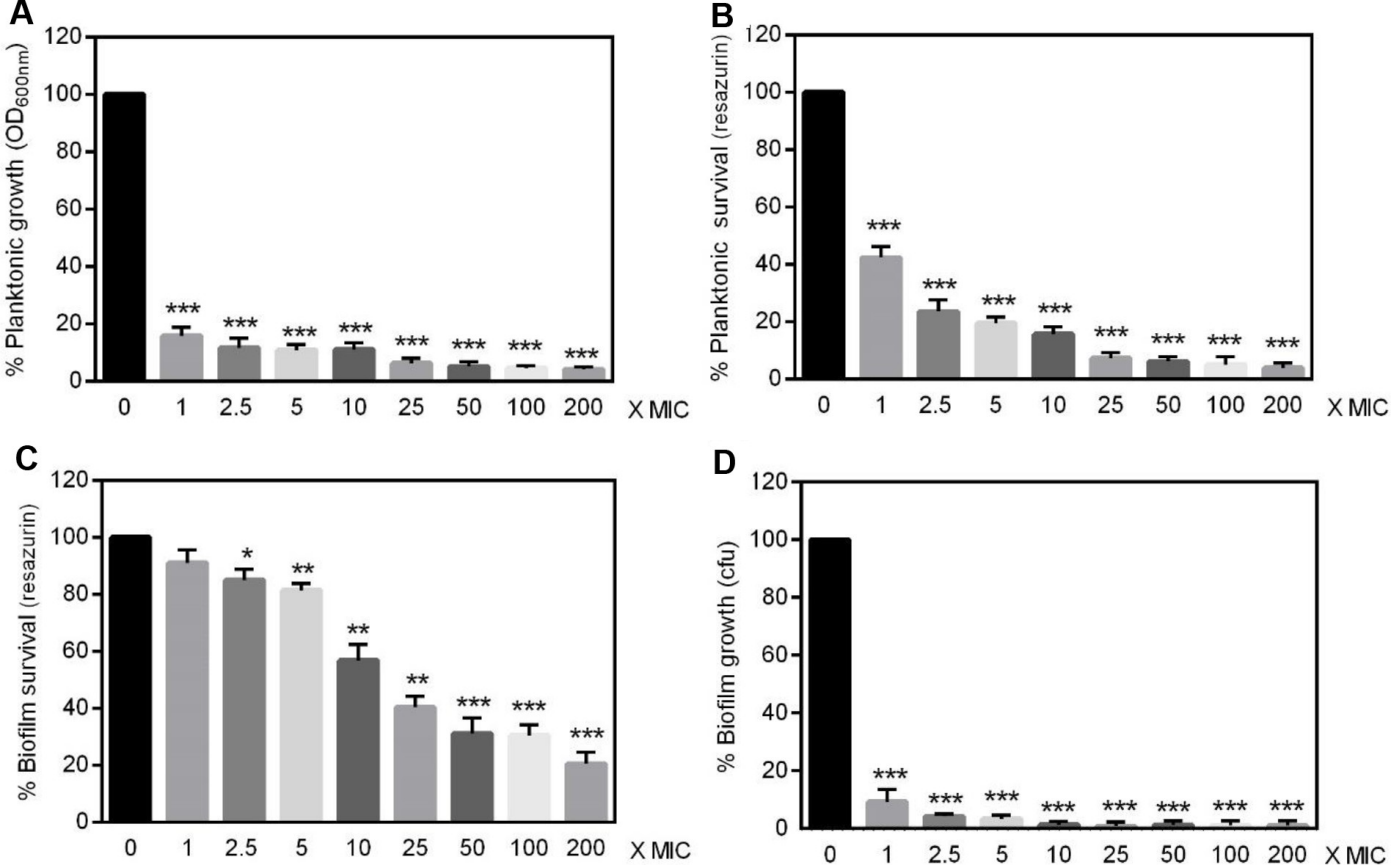
Ciprofloxacin susceptibility of *F*. *novicida* U112. **(A, B)** Planktonic or **(C, D)** 24 h-old biofilm bacteria were incubated for 24 h with concentrations of ciprofloxacin ranging from 1-time to 200-times the MIC (0.064 mg/L). **(A)** Bacterial replication in liquid media calculated from the OD_600nm_ **(B, C)** metabolic activity estimated using the resazurin reduction assay **(D)** amount of viable bacteria present in biofilms as determined by CFU counting. Values were expressed as percentage of data obtained without antibiotics and correspond to mean ± SEM of 6 replicates. Similar data were obtained in four to six experimental repeats.*P<0.05 **P<0.005 *** *P*< 0.0005.

Because bacteria embedded within biofilm matrix were often described extremely resistant to antibiotics [7, 8], we then compared the antibiotic susceptibility of *F*. *novicida* and *F*. *philomiragia* embedded in biofilms with that of planktonic cells. Two bactericidal antimicrobials commonly approved for the treatment of tularemia were used, i.e. ciprofloxacin and gentamicin [46]. The growth of planktonic cells exposed to antibiotics was determined from OD_600nm_ while their metabolic activity was estimated through the reduction of resazurin [39, 40]. This approach also conveniently enabled assessing the viability of biofilm bacteria whose survival was evaluated in parallel by CFU counting, as recently described for *F*. *tularensis* [16]. The results obtained demonstrated that *F*. *novicida* became significantly less susceptible to ciprofloxacin when growing within biofilms (Fig 7). Thus, approximately 40-times more ciprofloxacin was required to induce 80% reduction of the metabolic activity in biofilms relative to planktonic cultures (from 5x to 200x MIC) (Fig 7B and 7C). Strikingly, as few as 9.2% ± 2.4% bacteria (*n* = 3) were recovered from biofilms treated with the ciprofloxacin MIC (Fig 7D), whereas the bacterial viability evaluated in parallel through metabolic activity measurement was of 91.1% ± 2.7% (*n* = 3; *P*<0.0005) (Fig 7C).

**Fig 8:**
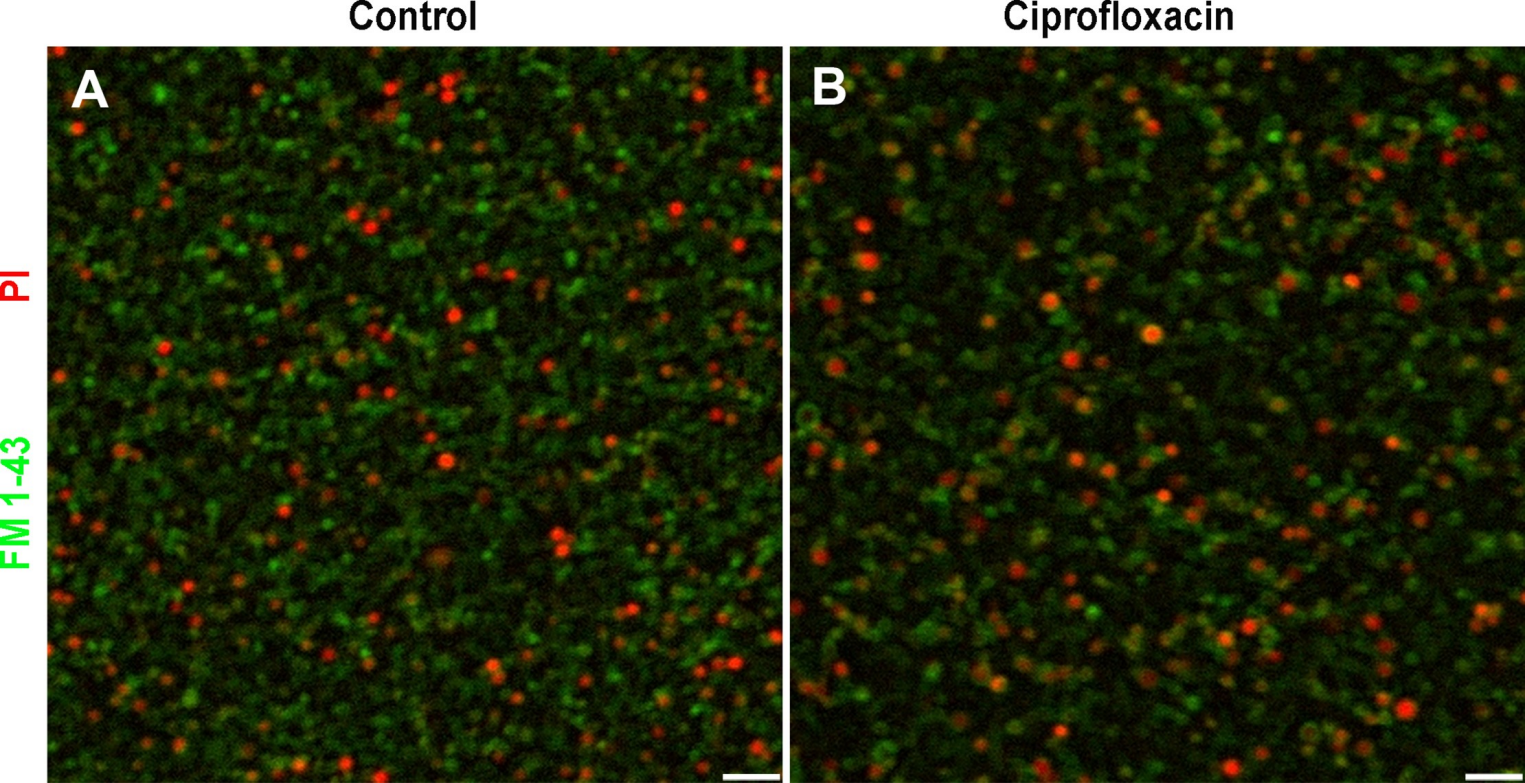
*F*. *novicida* in biofilms acquire a VBNC state after exposure to ciprofloxacin. Viability of *F*. *novicida* was analyzed in 24 h-old biofilms washed and incubated **(A)** in fresh MMH or **(B)** in presence of ciprofloxacin (0.32 mg/L; MIC 5x). Live and dead cells were visualized following 24 h incubation at 37°C by staining with the FilmTracer^™^ FM® 1–43 green biofilm cell stain (Molecular probes) and PI. The proportion of dead bacteria with damaged membranes exhibiting red fluorescence is similar in both conditions.

These data suggested that, in addition to the increased biofilm-specific antibiotic resistance, ciprofloxacin treatment induced a viable but non culturable (VBNC) state in *F*. *novicida* when organized as biofilm, similarly to what was reported for *F*. *tularensis* LVS [16]. The assumption that *F*. *novicida* enter a VBNC state in biofilm exposed to ciprofloxacin was further confirmed using the cell non-permeant DNA intercalating dye propidium iodide (Fig 8).

**Fig 9:**
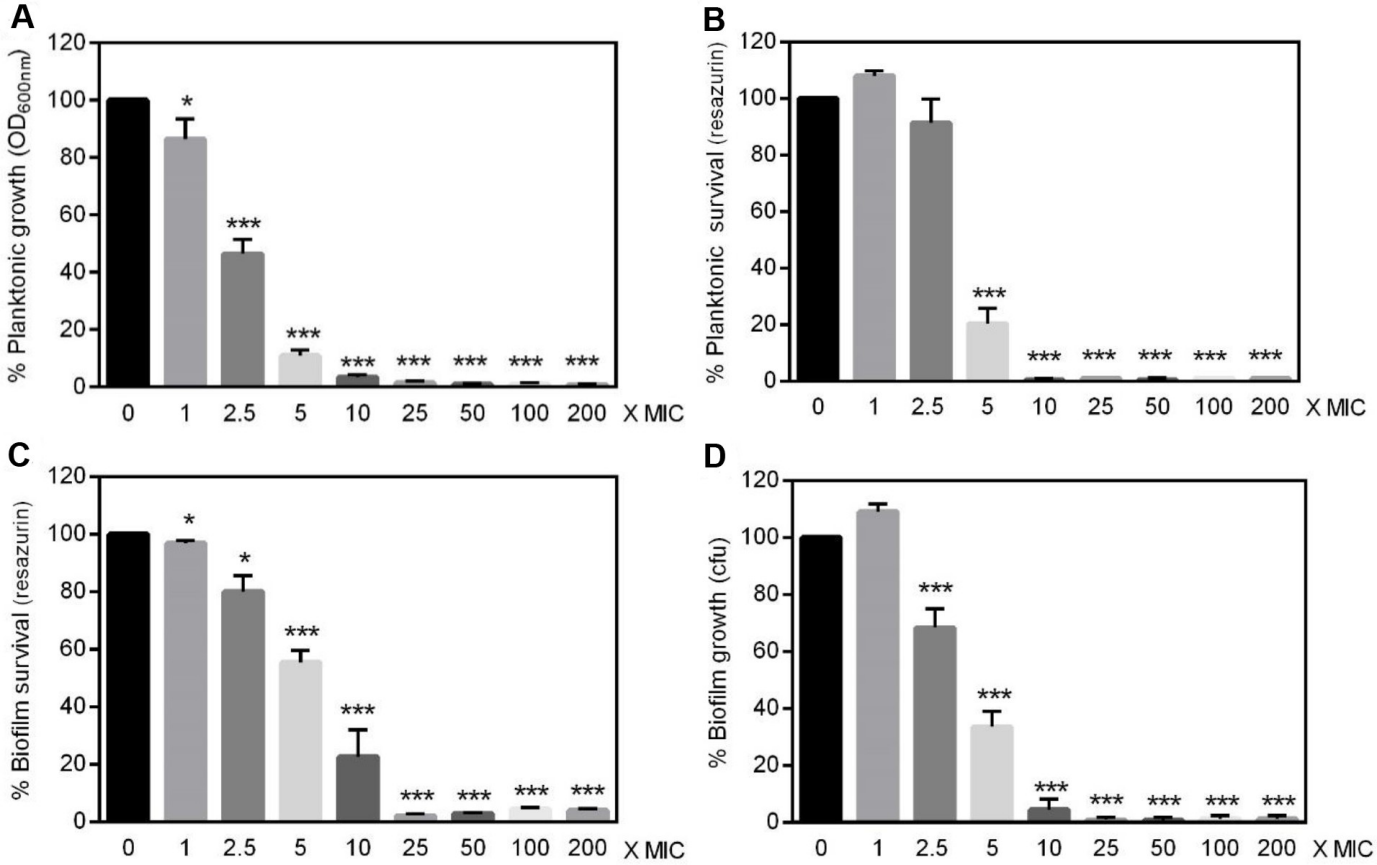
Gentamicin susceptibility of *F*. *novicida* U112. **(A, B)** Planktonic or **(C, D)** 24 h-old biofilm bacteria were incubated for 24 h with concentrations of gentamicin ranging from 1-time to 200-times the MIC (1 mg/L). **(A)** Bacterial replication in liquid media calculated from the OD_600nm_ **(B, C)** metabolic activity estimated using the resazurin reduction assay **(D)** amount of viable bacteria present in biofilms as determined by CFU counting. Values were expressed as percentage of data obtained without antibiotics and correspond to mean ± SEM of 6 replicates. Similar data were obtained in four to six experimental repeats.*P<0.05 **P<0.005 *** *P*< 0.0005.

**Fig 10:**
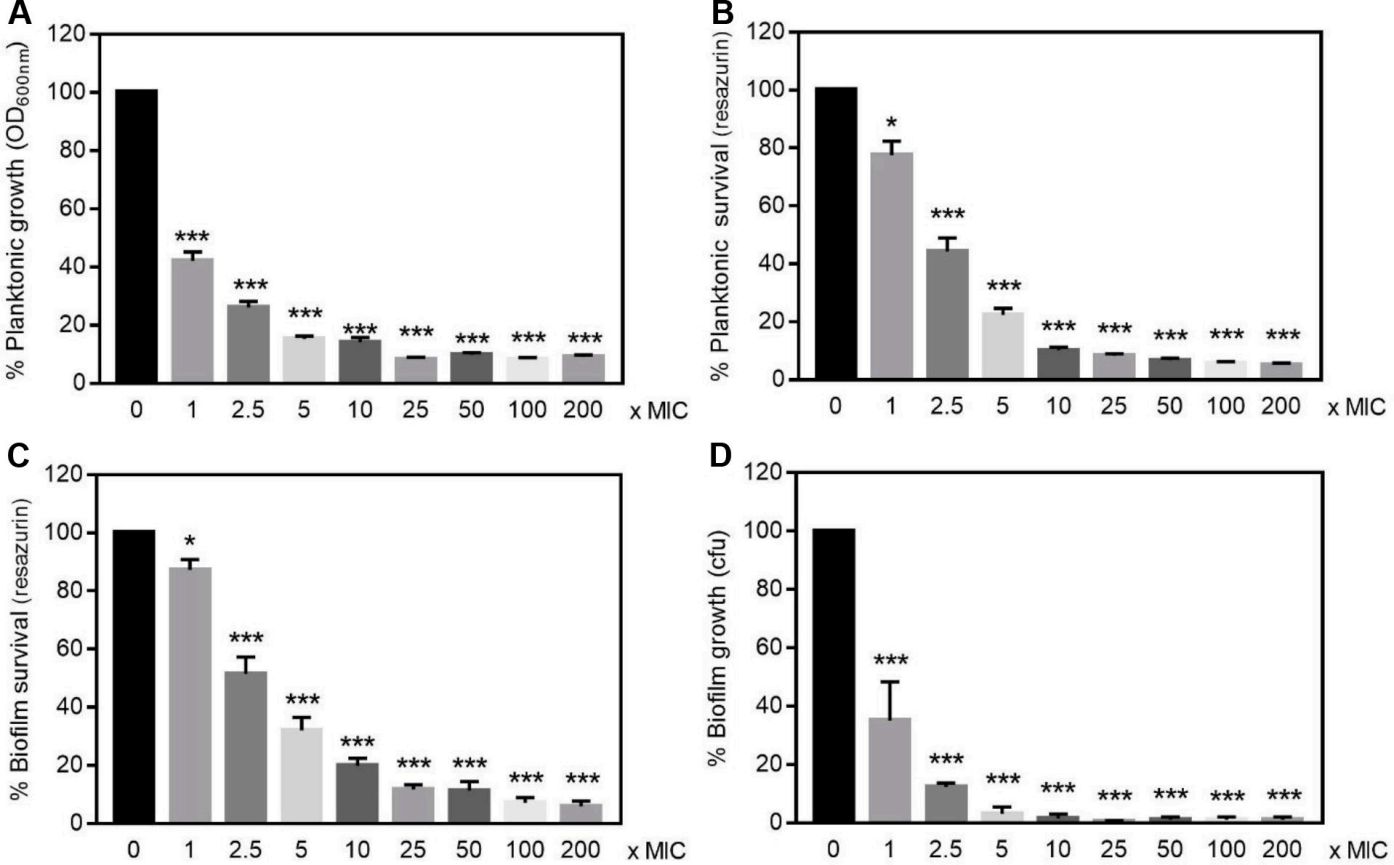
Ciprofloxacin susceptibility of *F*. *philomiragia* CHUGA-FP47. Same legend as in Fig 7 using 48h-old biofilm with a ciprofloxacin MIC value of 0.032 mg/L.

**Fig 11:**
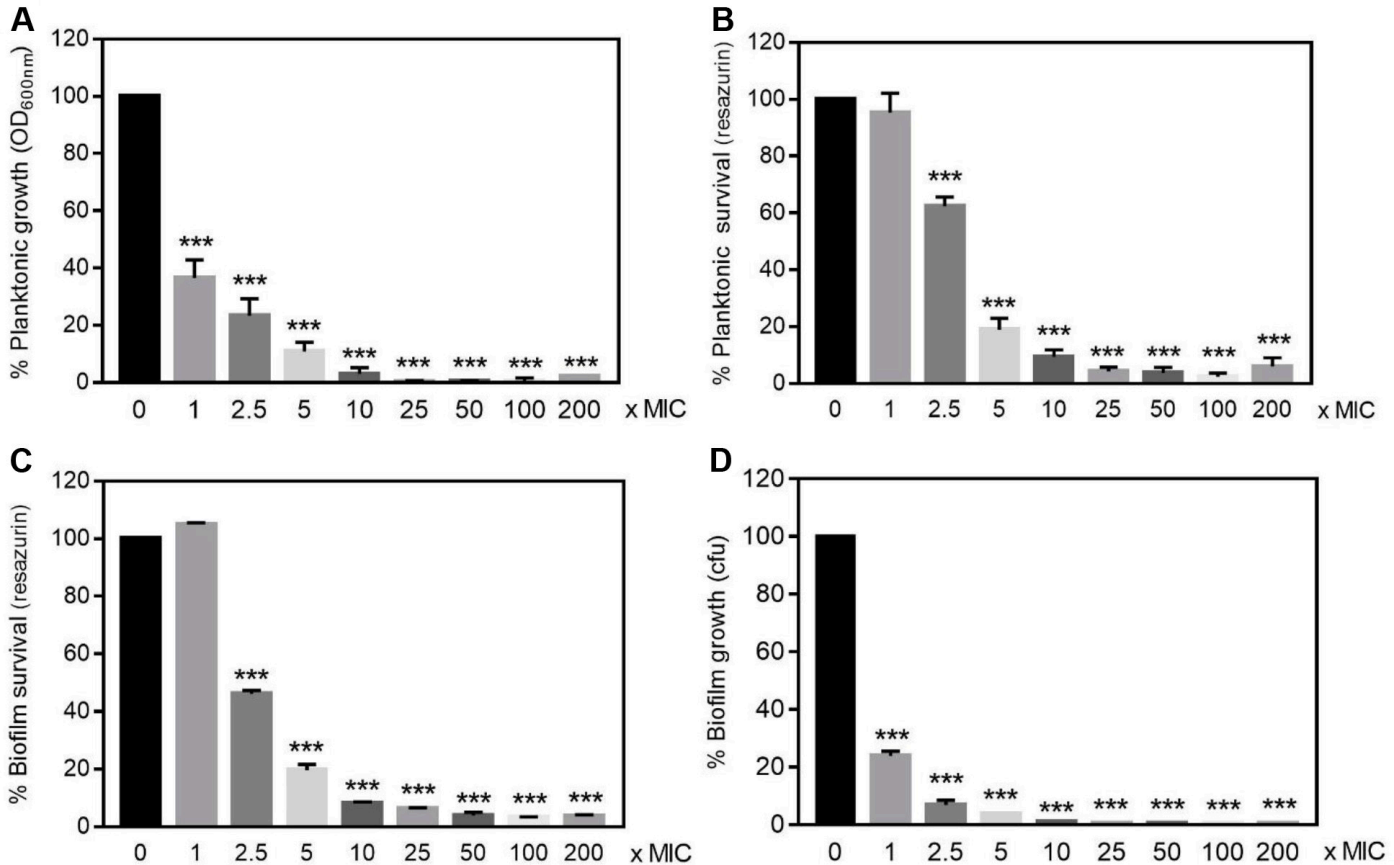
Gentamicin susceptibility of *F*. *philomiragia* CHUGA-FP47. Same legend as in Fig 8 using 48h-old biofilm with a gentamicin MIC value of 0.5 mg/mL.

This protective effect of *F*. *novicida* biofilm toward antibiotic was found specific for ciprofloxacin since gentamicin killed biofilm bacteria almost as efficiently as planktonic ones (Fig 9). Moreover, a significant correlation between the metabolic activity and CFU counting recovered from *F*. *novicida* biofilms exposed to gentamicin was observed (80.06% ± 3.2% *vs* 68.33% ± 9.8% respectively for 2.5x MIC; *n* = 3; *P* > 0.05), therefore confirming the reliability of resazurin fluorescence signal to quantitatively assess bacteria viability (Fig 9C and 9D). Interestingly, when embedded in biofilms, *F*. *philomiragia* did not acquire higher resistance than planktonic cells toward neither ciprofloxacin nor gentamicin (Figs 10 and 11).

### Survival of planktonic and biofilm-embedded *Francisella* in water

**Fig 12:**
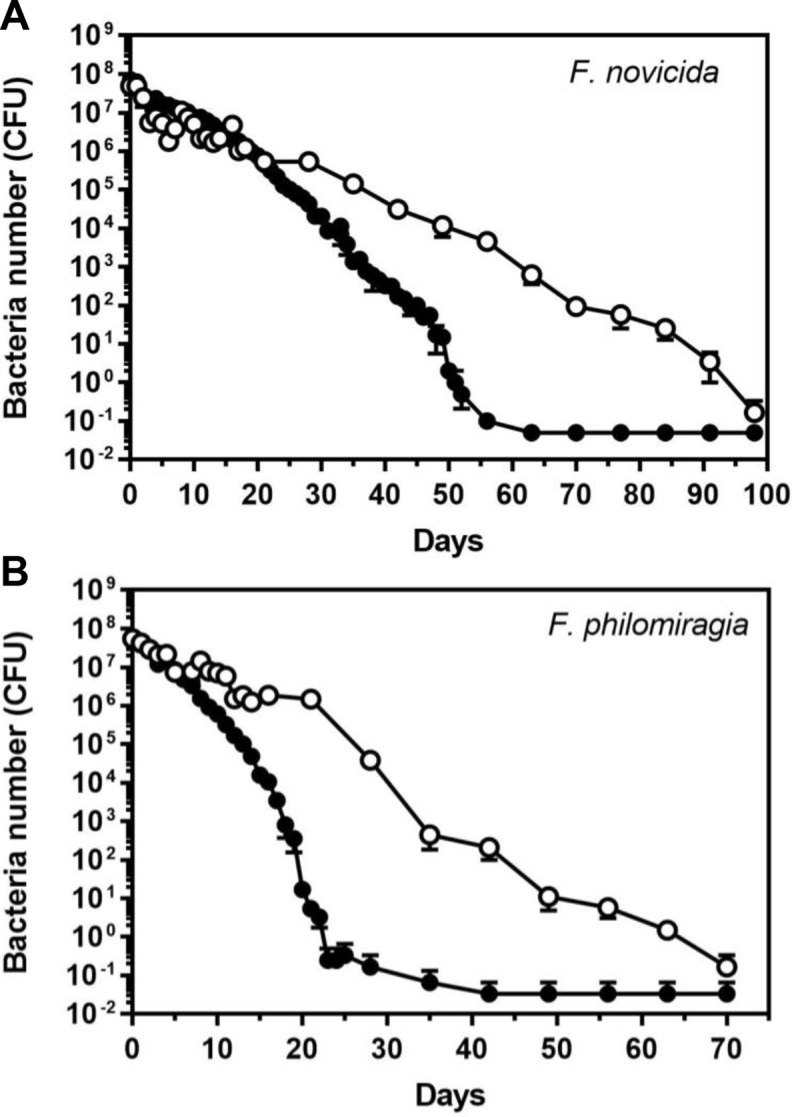
Survival of planktonic and biofilm bacteria in water. Exponential phase (black circles) or biofilm bacteria (white circles) were incubated in spring water at 4°C. Samples were taken every 24 h then every week and plated onto PVX-CHA plates to estimate the number of bacterial CFU. Data correspond to mean ± SEM of five independent cultures.

By providing the capacity of bacteria to survive in nutrient-limited aquatic environment, the biofilms could also contribute to the environmental persistence and thus indirectly promote the transmission of *Francisella* to hosts [13, 23]. To adress this question, *Francisella* biofilms formed in microtiter plates were incubated in water at 4°C and survival of bacterial was evaluated over time by CFU counting until no bacteria were recovered. These viability scores were compared with those of exponential growth phase (planktonic) bacteria at similar density in water and thus exposed to the same conditions. Interestingly, the obtained CFU decay curve of planktonic *F*. *novicida* in spring water (Fig 12A) was found comparable to that observed by Berrada and Telford when incubating the same strain in brackish-water [22]. Importantly, our results showed that both strains survived longer to cold water exposure when embedded in biofilms. Thus, while planktonic *F*. *novicida* persisted no more than nine weeks, at this stage 1.18 x10^4^ ± 0.5 x10^4^ (*n* = 6) colonies were still enumerated from the biofilm. Actually, *F*. *novicida* survived two times longer in biofilm than their planktonic counterparts (52 *vs* 98 days) (Fig 12A). Importantly, the biofilm was found to extend even more significantly the survival of the clinical strain *F*. *philomiragia* CHUGA-FP47 from three to seven weeks in cold water (Fig 12B).

## Discussion

In this study, we bring new insights on *Francisella* biofilms using the strains *F*. *novicida* and *F*. *philomiragia* for which human infections have been mainly associated with environmental contaminations including near-drowning events and use of outdoor ice machines [29–31]. In contrast to the highly virulent *F*. *tularensis* strains that can only be assayed under BSL-3 conditions, these strains can be handled under standard BSL-2 laboratory, being thus more tractable to live imaging. The selection of these strains in this study was also driven by their capacity to form higher biofilm biomasses than the *F*. *tularensis* subspecies *tularensis* (SCHU S4) [3] or *F*. *tularensis* subspecies *holarctica* LVS [3, 16], hence being more relevant to assess how dispersing agents impact on bacteria viability.

To ensure reproducible biofilm assays, we first evaluated the formation of the biofilm biomass relative to the initial number of bacteria. To assess the biofilm matrix composition while reducing potential bias due to heterogeneity in nutrient availability, a limitation already described for *Staphylococcus aureus* [47] and also recently for *Francisella* [48], both strains were grown in MMH at 37°C. Under such conditions, we determined that the biofilm development by *F*. *novicida* was optimal starting from a bacterial inoculum of 10^7^ CFU/mL, this value being of 5.10^7^ bacteria/mL for *F*. *philomiragia*. Live and static images analyzed over time confirmed a slower kinetic of bacterial replication and assembly of *F*. *philomiragia* biofilm as compared to *F*. *novicida* as previously observed when comparing other *F*. *novicida* and *F*. *philomiragia* isolates grown at 37°C [4]. It can be hypothesized that different capacity of both strains to form biofilms could result from a differential activation of genes encoding two-components system or of quorum sensing involved in the maturation and disassembly of biofilms [49, 50]. Immunofluorescence confocal microscopy also provided structural insights of the mature biofilms. Interestingly, as early as 2 h of growth under static conditions, the cocobacillus shape typically observed for exponentially growing *F*. *novicida* was lost and the bacteria shifted to a coccoid form. Changes of bacterial shapes are not accidental but are rather described as biologically relevant [51]. Indeed the coccoid morphology previously observed for *F*. *novicida* biofilms grown on chitin surfaces [3], but never reported before for *F*. *philomiragia*, could reflect lower nutrient requirements and slow-growth conditions resulting from oxygen limitation under static conditions. Both strains, which secreted EPS containing glucose and mannose residues, as shown by ConA-FITC binding, later organized into a several micron-sized 3D structure. The thickness of *F*. *novicida* biofilm was quantified by CLSM by others [3, 14, 48], but the comparison of results obtained across different laboratories is challenging when considering differences in nutrient availability, temperature, or the flow conditions [52]. In this study, a slight discrepancy between biofilm thickness and their biomass assessed with crystal violet staining was noticed and could result from the growth devices respectively used (coated glass coverslips or polystyrene microtiter plates) [52]. Of note, the detachment or erosion of the structurally fragile *F*. *philomiragia* biofilm provoked by extensive washings during immunostaining procedure cannot be excluded.

Based on the resulting biofilm morphology, 24 h-old *F*. *novicida* and 48 h-old *F*. *philomiragia* biofilms were respectively used to determine their composition and to investigate their potential protective effect against chemical stresses or cold water exposure mimicking the aquatic environmental niche of these microorganisms. Results obtained highlighted marked differences in the composition of the biofilm between *F*. *novicida* U112 and *F*. *philomiragia* CHUGA-FP47. Only DNase I and EDTA significantly reduced the biofilm biomass of both strains, suggesting that eDNA is a major common structural component of *Francisella* biofilms, similarly to several species of bacteria [53]. Surprisingly, the biofilm produced by *F*. *novicida* was found more susceptible than the *F*. *philomiragia* biofilm to almost all the dispersing agents tested despite a much higher mechanical resistance to fluids during washing procedures. Even more striking was the proteinase K resistance of the *F*. *philomiragia*-associated EPS that exceeds about four hundred times the *F*. *novicida*-associated matrix. Since proteinase K is a potent and broad*-*spectrum serine protease, these data indicated that the stabilization of *F*. *philomiragia* biofilm does not require proteinaceous proteinase K-sensitive adhesins although the contribution of proteins exhibiting another proteolytic profile [54] or resistant to proteases, like amyloid fibers [55], cannot be excluded. In addition to nucleic acids and proteins, exopolysaccharides including cellulose and polymeric β-1,4-linked *N*-acetylglucosamine were also identified within the biofilm matrix of *F*. *novicida*. These EPS components appear to be missing in *F*. *philomiragia* biofilm, which indeed remained intact after cellulase or chitinase treatments. The observed mechanical fragility of *F*. *philomiragia* biofilms fits well with the absence of the rigid β-1,4-linked polymers, notably cellulose [56]. Such « viscous hence non rigid mass » biofilms were demonstrated to be ecologically advantageous to *Pseudomonas* strains in a static liquid microcosm [56], a point supporting water source as potential reservoir for the clinical strain CHUGA-PFP47. Another phenotypic difference between both biofilms relies on their distinct sensitivity to dispersin B. Interestingly, the negative correlation between the dispersal efficacy of proteinase K and dispersin B that characterizes *F*. *philomiragia* biofilm is also shared for different isolates of *S*. *aureus* [47, 54].

Over the last decade, it became increasingly clear that the formation of biofilm was implicated in resistance towards antimicrobials and caused persistent infections for many bacterial species [5, 7, 8, 10]. Accordingly, in recent years several biofilm-targeting approaches were developed as emerging therapeutic strategies [57]. This bacterial lifestyle usually results in a lower efficacy of antimicrobials that need to penetrate the dense biofilm matrix, coupled to a slow or even stopped bacteria growth, hence protecting microbes from the detrimental effects of both antibiotics and the host immune system. Considering the diffusion barrier limit imposed by the EPS in the biofilms, the protection provided is intrinsincally linked to its biochemical composition as examplified by the interactions between gentamicin and alginate, the major component of mucoid *P*. *aeruginosa* biofilms [58]. Variations in the components ensuring the structural integrity of biofilms formed by different isolates of a bacterial strain such as *S*. *aureus* [47, 59] or *Propionibacterium acnes* [60] were previously reported. Accordingly, the heterogeneous composition of biofilms formed by *F*. *novicida* or *F*. *philomiragia* observed here is not surprising. This biofilm structural heterogeneity was found associated with specific and unexpected responses towards exogenous stressors including exposure to antibiotics and cold water. Indeed *F*. *novicida* biofilms displayed a significantly lower susceptibility towards ciprofloxacin than planktonic bacteria, hence raising important concerns about current therapies as this antibiotic belongs to the first-line agents to treat tularemia [1, 46]. Such a ciprofloxacin-protected lifestyle was also recently observed with a *F*. *tularensis* LVS isolate [16]. In contrast, the clinical strain *F*. *philomiragia* CHUGA-FP47 showed unaltered antibiotic resistance regardless of the free or biofilm-associated bacteria status.

Besides protecting bacteria against antibiotics, the biofilm formation by *Francisella* spp. was hypothesized to be key mechanism of environmental survival and persistence, specifically in aquatic habitats which constitute an important ecological niche of these microorganisms [13, 23, 61]. In line with this scheme, we now report that both strains growing in biofilms were far more resistant to harsh water-based conditions than free-living bacteria, an effect particularly pronounced for the clinical strain of *F*. *philomiragia* CHUGA-FP47.

In summary, our findings pave the way for further in-depth investigations to optimize the design of new therapeutic approaches against tularemia in particular with the combined use of antibiotics and dispersal agents or antibiotic adjuvants [7]. In this context, the precise role of eDNA, a common feature of *Francisella* EPS, in biofilm formation and stability should be thoroughly addressed. However, our data already indicate that treatment of these biofilms with DNase I and EDTA, two compounds already approved for human use [62, 63] may enhance the ability of antibiotics to clear infections, especially when ciprofloxacin is used. Finally, this study suggests that the functional role of biofilms could be intrinsically linked to their EPS composition with a proteinase K resistant and carbohydrate-rich matrix [54] hence more prone to promote the survival of *Francisella* spp. in aquatic environments.

## Supporting information

S1 Movie Formation of biofilm by *F*. *novicida* and *F*. *philomiragia*. Videorecording of bacterial suspension grown at 37°C 5% CO_2_ without agitation in Chamlide^™^ chambers installed on an Eclipse Ti inverted. DIC images were taken at 15 min interval over a 17h50 and 21h20 period for *F*. *novicida* and *F*. *philomiragia*, respectively. Scale bar: 5 μM.
(AVI)
